# Supplementary material for: Site-specific cleavage of bacterial MucD by secreted proteases mediates antibacterial resistance in Arabidopsis
Source: Nat Commun. 2019 Jun 28;10:2853. doi: 10.1038/s41467-019-10793-x (PMC6599210; doi:10.1038/s41467-019-10793-x)
Supplement: Supplementary file 1 — Supplementary Information [file 41467_2019_10793_MOESM1_ESM.pdf]

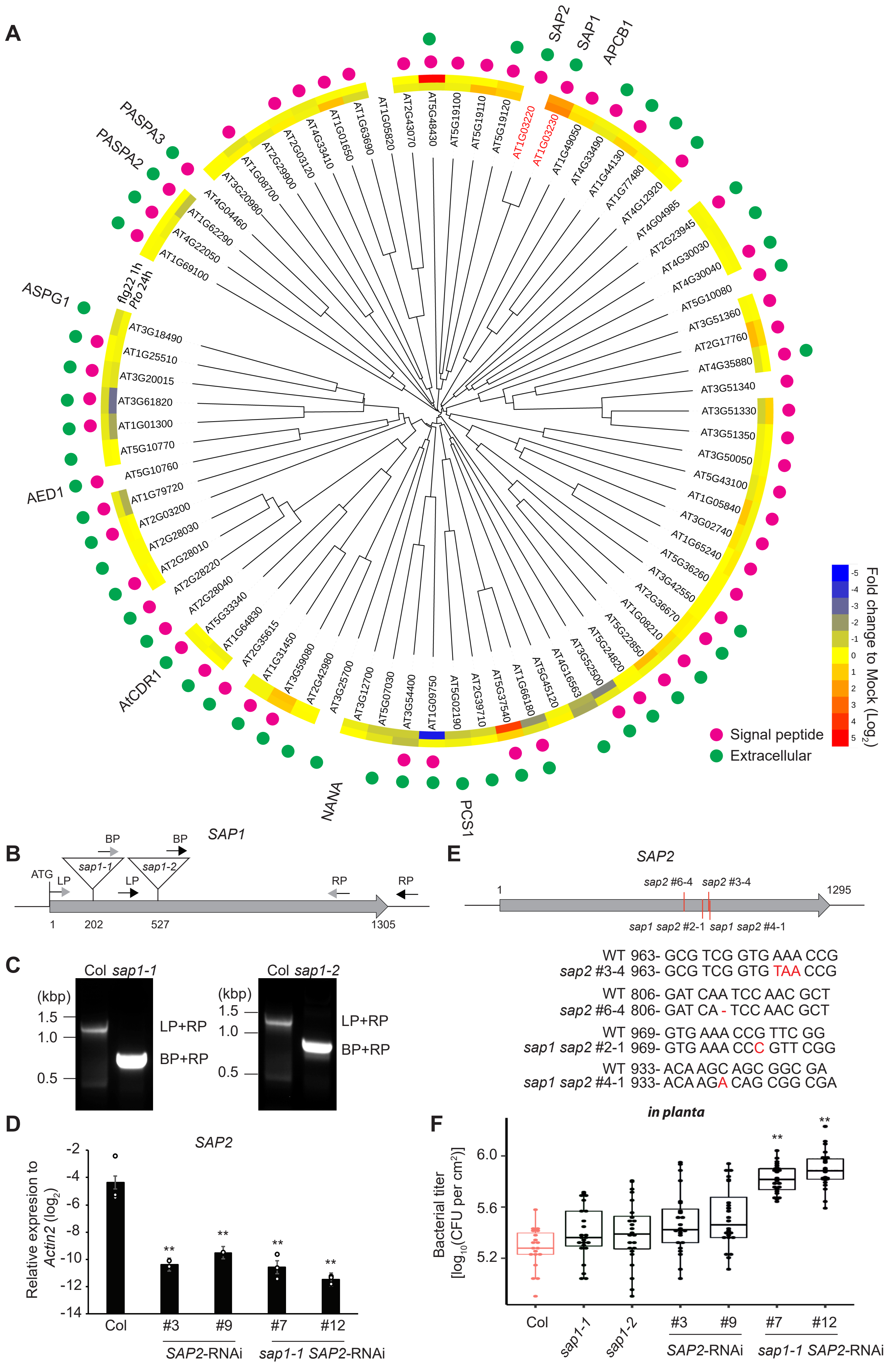
A
SAP2
SAP1
APCB1
PASPA3
PASPA2
ASPG1
AED1
AtCDR1
NANA
PCS1
flg22 1h
Pto 24h
AT1G05820
AT2G43070
AT5G48430
AT5G19100
AT5G19110
AT5G19120
AT1G03220
AT1G03230
AT1G49050
AT4G33490
AT1G44130
AT1G77480
AT4G12920
AT4G04985
AT2G23945
AT4G30030
AT4G30040
AT5G10080
AT3G51360
AT2G17760
AT4G35880
AT3G51340
AT3G51330
AT3G51350
AT3G50050
AT5G43100
AT1G05840
AT3G02740
AT1G65240
AT5G36260
AT3G42550
AT2G36670
AT1G08210
AT5G22850
AT5G24820
AT3G52500
AT4G16563
AT5G45120
AT1G66180
AT5G37540
AT2G39710
AT5G10760
AT1G09750
AT3G54400
AT5G07030
AT3G12700
AT3G25700
AT2G42980
AT3G59080
AT1G31450
AT2G35615
AT1G64830
AT5G33340
AT2G28040
AT2G28220
AT2G28010
AT2G28030
AT2G03200
AT1G79720
AT5G10760
AT5G10770
AT1G01300
AT3G61820
AT3G20015
AT1G25510
AT3G18490
AT1G69100
AT4G22050
AT1G62290
AT4G04460
AT3G20980
AT1G08700
AT2G29900
AT2G03120
AT4G33410
AT1G01650
AT1G63690
Fold change to Mock (Log2)
-5
-4
-3
-2
-1
0
1
2
3
4
5
Signal peptide
Extracellular
B
SAP1
ATG
LP
BP
sap1-1
sap1-2
RP
1
202
527
1305
C
Col sap1-1
Col sap1-2
(kbp)
1.5
1.0
0.5
LP+RP
BP+RP
D
SAP2
Relative expression to Actin2 (log2)
-2
-4
-6
-8
-10
-12
-14
**
Col
#3
#9
#7
#12
SAP2-RNAi
sap1-1 SAP2-RNAi
E
SAP2
1
1295
sap2 #6-4
sap2 #3-4
sap1 sap2 #2-1
sap1 sap2 #4-1
WT 963- GCG TCG GTG AAA CCG
sap2 #3-4 963- GCG TCG GTG TAA CCG
WT 806- GAT CAA TCC AAC GCT
sap2 #6-4 806- GAT CA - TCC AAC GCT
WT 969- GTG AAA CCG TTC GG
sap1 sap2 #2-1 969- GTG AAA CCC GTT CGG
WT 933- ACA AGC AGC GGC GA
sap1 sap2 #4-1 933- ACA AGA CAG CGG CGA
F
in planta
Bacterial titer [log10(CFU per cm2)]
6.0
5.6
5.2
**
Col
sap1-1
sap1-2
#3
#9
#7
#12
SAP2-RNAi
sap1-1 SAP2-RNAi

**Supplementary Fig. 1** Characterization of the T-DNA insertion mutants, RNAi lines, and CRISPR-Cas9 lines for *SAP1* and *SAP2*. **A** Amino acid sequences of 77 aspartic proteases annotated in the MEROPS database (https://merops.sanger.ac.uk/) were subjected to phylogenetic analysis using CLUSTALW (http://www.genome.jp/tools/clustalw/). Gene expression profiles upon flg22 treatment at 1 hpi and *Pto* infection at 24 hpi were obtained from Genevestigator and a heatmap was generated by iTOL[1, 2]. Blue and red indicate suppression and induction compared to control, respectively. Gene names were given when they were previously reported or are described in this study. Signal peptides and extracellular localization were predicted by SignalP4.1 and TAIR10 web tools, respectively. **B** Schematic diagram of T-DNA insertion lines for *SAP1*. Gray and black arrows indicate the primers used for genotyping *sap1-1* and *sap1-2*, respectively. **C** Genotyping of *sap1-1* and *sap1-2* homozygous mutants by PCR using genomic DNA. **D** Expression of *SAP2* in *SAP2 RNAi* and *sap1-1 SAP2 RNAi* lines by RT-qPCR. Bars represent means and s.e.m of three biological replicates. The vertical axis shows the $\log_2$ expression levels relative to *Actin2*. Asterisks indicate significant differences from Col (Student's two-tailed t-test; ** P<0.01). **E** Schematic diagram of *sap2* CRISPR-Cas9 mutants. Red bars indicated mutation sites in *SAP2*. Detailed sequence changes in individual lines are listed in red. **F** Leaves of four-week-old Col, *sap1-1*, *sap1-2*, *SAP2-RNAi,* and *sap1-1 SAP2-RNAi* plants were infiltrated with *Pto* ($OD_{600}$ = 0.0005), and bacterial titer was determined at 3 dpi. Bars represent means and s.e.m of four independent experiments with four biological replicates. Asterisks indicate significant differences from Col (Student's two-tailed t-test; ** P<0.01).

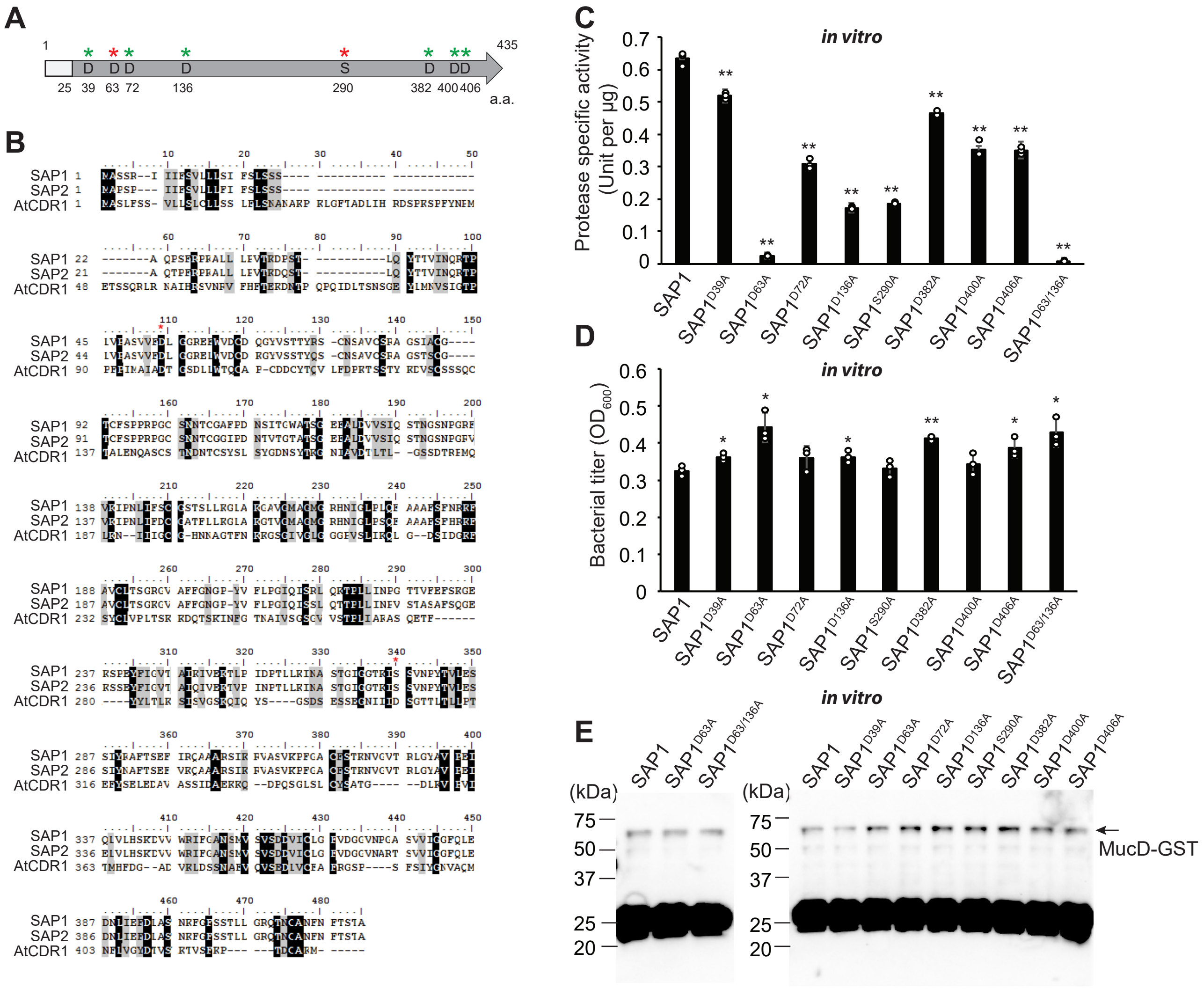


**Supplementary Fig. 2** Site-directed mutagenesis of SAP1. **A** Schematic diagram of site-directed mutagenesis of SAP1. The aspartic acid (D) and serine (S) residues at different positions were replaced with alanine (A). **B** Amino acid sequence alignment of *A. thaliana* CDR1 (AtCDR1), SAP1, and SAP2. Red asterisks indicate two Asp residues in the active site of AtCDR1. **C** Protease activity of recombinant wild-type and mutated SAP1 proteins was measured. Bars represent means and s.e.m of three biological replicates. Asterisks indicate significant differences from the wild type SAP1 (Student's two-tailed t-test; ** P<0.01). **D** *In vitro* growth of *Pto* ($OD_{600}$ = 0.005) was measured 6 h after treatment with recombinant proteins. Bars represent means and s.e.m of three biological replicates. Asterisks indicate significant differences from the wild type SAP1 (Student's two-tailed t-test; * P<0.05, ** P<0.01). **E** Recombinant SAP1 proteins were detected by immunoblotting using an anti-GST antibody.

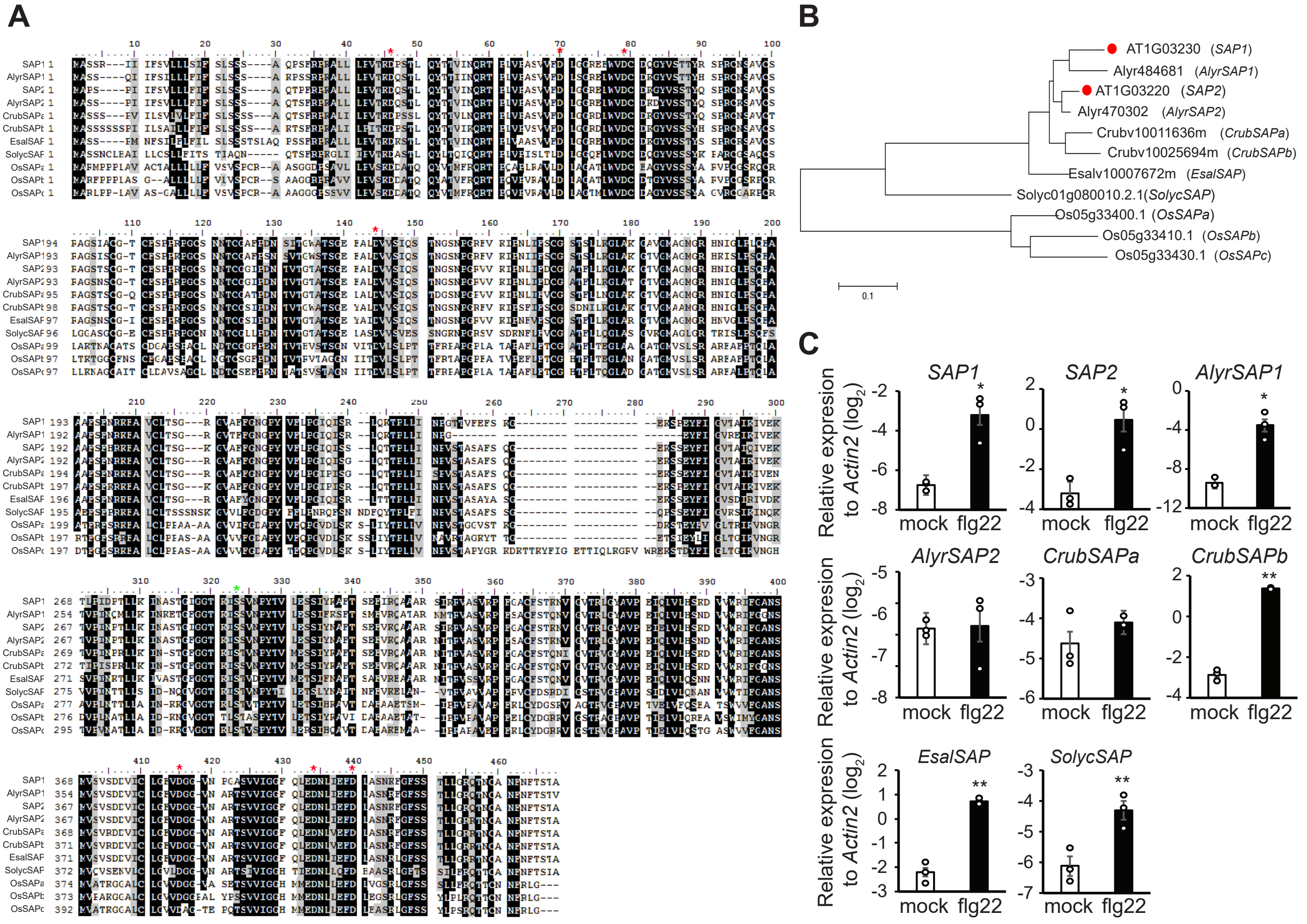


**Supplementary Fig. 3** Conservation of SAP1 homologs in different plant species. **A** Amino acid sequence alignment of SAP1 homologs in plants, including *Arabidopsis lyrata*, *Capsella rubella*, *Eutrema salsugineum*, *Solanum lycopersium*, and *Oryza sativa*. Red and green asterisks indicate mutated Asp and Ser residues as in Supplementary Fig. 2A. **B** Amino acid sequences of SAP1 and SAP2 homologs from different plant species, including *Capsella grandiflora*, *C. rubella*, *A. lyrata*, *Brassica rapa*, *E. salsugineum*, *S. lycopersium*, and *O. sativa*, were obtained from Phytozome (https://phytozome.jgi.doe.gov/pz/portal.html) and subjected to phylogenetic analysis. **C** Expression of *SAP1* homologs was determined by RT-qPCR in 12-days-old seedlings of *C. rubella*, *A. lyrata*, *E. salsugineum*, and *S. lycopersicum* at 1 h after treatment with 1 μM flg22 or mock. Bars represent means and s.e.m of three biological replicates. Asterisks indicate significant differences from mock (Student's two-tailed t-test; * $P<0.05$, ** $P<0.01$).

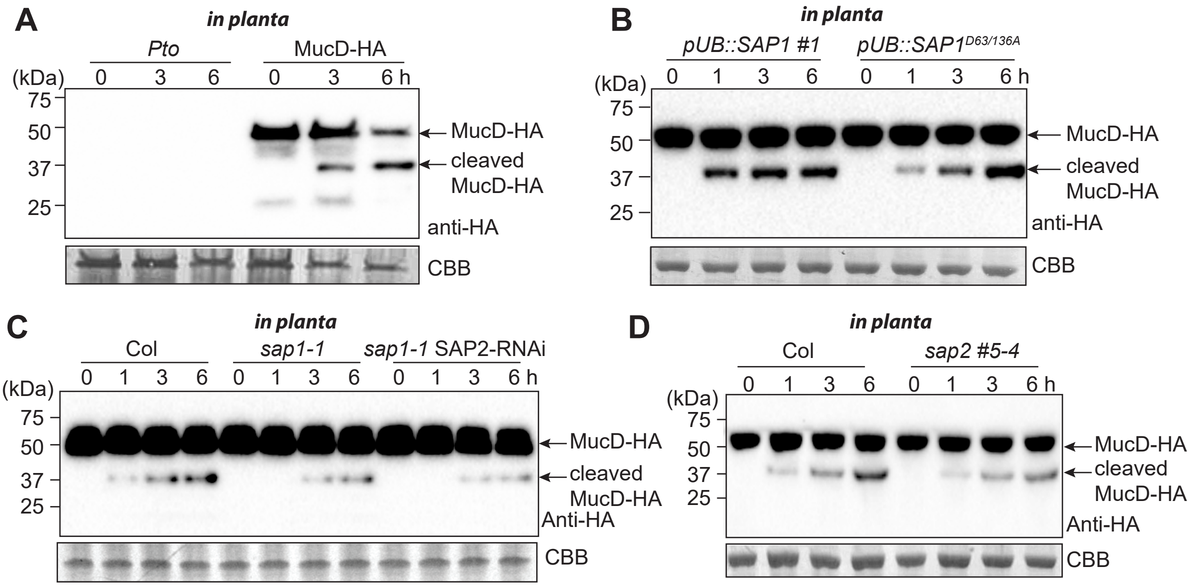


**Supplementary Fig. 4** MucD cleavage is linked to SAP1 and SAP2 activity *in planta*.
**A-D** Wild type *Pto* and *Pto* Δ*mucD* expressing MucD-HA ($OD_{600}$ = 0.05) were infiltrated into leaves of four-week-old Col, pUB::*SAP1-RFP*, pUB::*SAP1$^{D63/136A}$-RFP*, *sap1-1*, *sap1-1 SAP2-RNAi*, and *sap2* plants, and total protein was extracted at the indicated time points. MucD-HA was detected by immunoblotting using an anti-HA antibody. CBB-stained Rubisco large subunit serves as loading control.

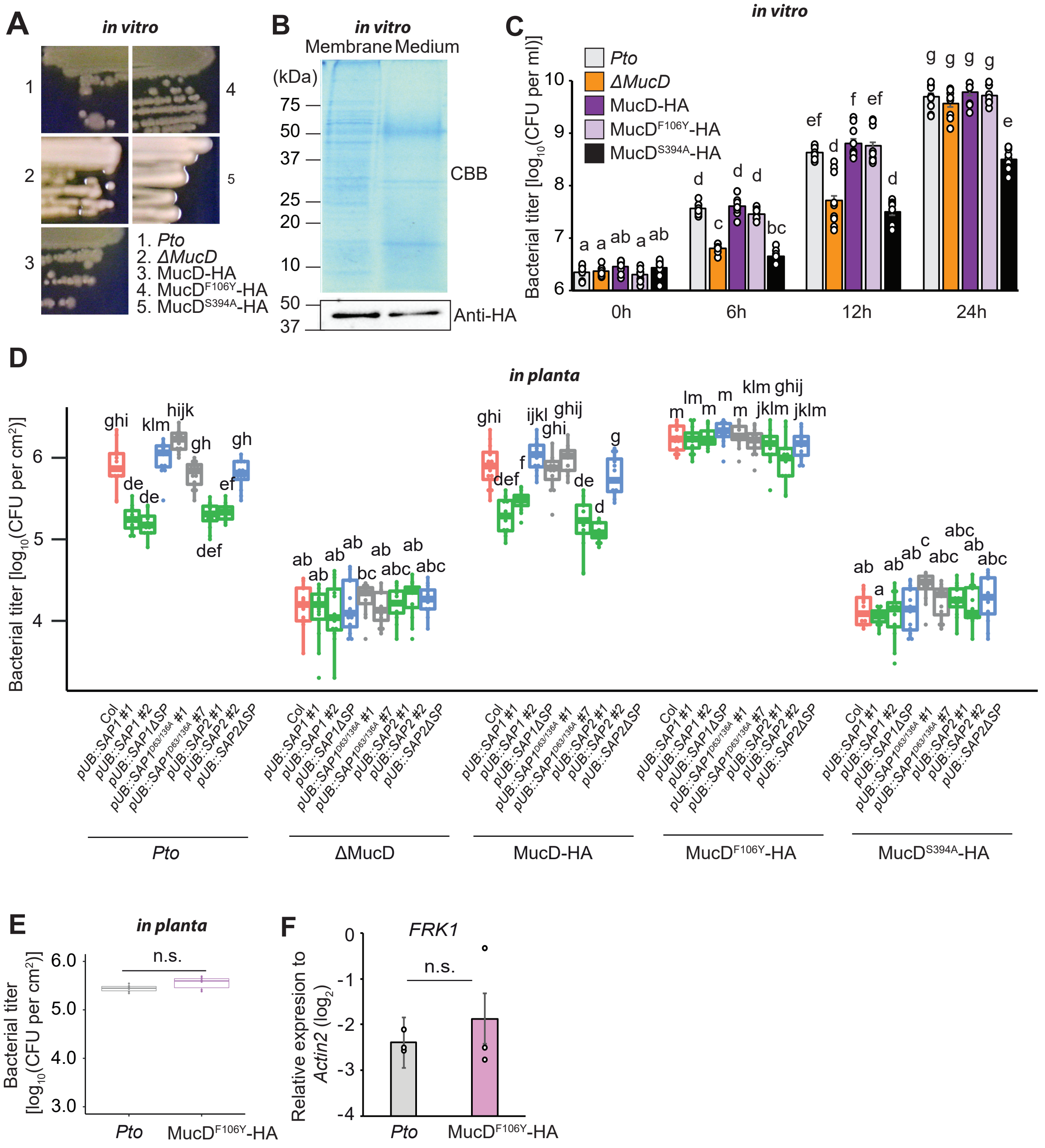


**Supplementary Fig. 5** SAP-mediated cleavage of MucD suppresses *Pto* growth. **A** Growth phenotype of *Pto*, *Pto ΔmucD*, or *Pto ΔmucD* expressing MucD-HA, MucD$^{F106Y}$-HA, or MucD$^{S394A}$-HA on King's B medium after 2 days at 28°C. **B** Proteins from the bacterial membrane and culture medium from *Pto ΔmucD* expressing MucD-HA were separated on SDS-PAGE and stained with CBB. MucD-HA was detected by immunoblotting using an anti-HA antibody. **C** *In vitro* growth in King's B medium with rifampicin ($OD_{600}$ = 0.005) over time. Data represent means and s.e.m of three independent experiments each with three biological replicates. **D** *Pto*, *Pto ΔmucD*, or *Pto ΔmucD* expressing MucD-HA, MucD$^{F106Y}$-HA, or MucD$^{S394A}$-HA ($OD_{600}$=0.001) were infiltrated into leaves of Col, pUB::*SAP1-RFP*, pUB::*SAP1ΔSP-RFP*, pUB::*SAP1$^{D63/136A}$-RFP*, pUB::*SAP2-RFP*, and pUB::*SAP2ΔSP-RFP* plants, and bacterial titer was determined at 2 dpi. Bars represent means and s.e.m of three independent experiments with at least three biological replicates. **C**, **D**, Statistically significant differences are indicated by different letters (Adjusted P<0.01). **E**, **F**, Bacterial titer (**E**) and relative expression of *FRK1* (**F**) in leaves of four-week-old Col plants infiltrated with *Pto* and *Pto ΔmucD* expressing MucD$^{F106Y}$-HA ($OD_{600}$ = 0.05) at 6 hpi. The vertical axis shows the $log_2$ expression levels relative to *Actin2*. Bars represent the means and s.e.m of two (**E**) or three (**F**) biological replicates. n.s., not significant (Student's two-tailed t-test).

**Supplementary Table 1. List of the identified proteins through LC-MS/MS analysis.**

| Number | Accession | Name | Meta Score A | Meta Score B | Meta Score C | Peptides A | Peptides B | Peptides C | sequence converage [%] A | sequence converage [%] B | sequence converage [%] C | Molecular Mass (Da) | Aspartic protease digestion site |
|---|---|---|---|---|---|---|---|---|---|---|---|---|---|
| 1 | PSPTO_0425 | Zn-dependent peptidases | 1013.0 | 514.6 | 986.3 | 16 | 8 | 14 | 36.6 | 16.9 | 35.8 | 52.29 | 0 |
| 2 | PSPTO_4221 | serine protease, MucD | 912.5 | 542.9 | 1120.1 | 17 | 12 | 20 | 43.2 | 17.5 | 55.1 | 50.37 | 2 |
| 3 | PSPTO_0369 | outer membrane porin, OprD family | 870.8 | 468.0 | 964.1 | 12 | 7 | 16 | 31.3 | 16.6 | 34.0 | 48.53 | 0 |
| 4 | PSPTO_4861 | carboxylase, biotin carboxylase | 329.3 | 182.3 | 572.8 | 5 | 3 | 10 | 16.8 | 8.9 | 29.3 | 48.57 | 1 |
| 5 | PSPTO_3727 | Trigger factor | 238.1 | 93.8 | 336.3 | 2 | 1 | 5 | 5.0 | 2.3 | 14.7 | 48.64 | 3 |
| 6 | PSPTO_4664 | RNA helicase rhlE | 188.9 | 0.0 | 133.2 | 2 | 0 | 2 | 4.8 | 0.0 | 5.4 | 48.68 | 4 |
| 7 | PSPTO_1296 | porin B | 137.3 | 0.0 | 178.8 | 2 | 0 | 3 | 5.1 | 0.0 | 9.9 | 50.30 | 0 |
| 8 | PSPTO_5560 | TonB-dependent receptor | 115.6 | 0.0 | 112.7 | 3 | 0 | 2 | 6.8 | 0.0 | 2.8 | 81.66 | 2 |
| 9 | PSPTO_4171 | amino acid ABC transporter substrate-binding protein | 110.0 | 0.0 | 56.6 | 2 | 0 | 1 | 7.1 | 0.0 | 3.9 | 33.51 | 1 |
| 10 | PSPTO_4561 | dipeptide ABC transporter substrate-binding protein | 100.5 | 0.0 | 94.8 | 2 | 0 | 2 | 4.0 | 0.0 | 4.0 | 59.14 | 2 |
| 11 | PSPTO_2173 | 3-isopropylmalate dehydratase large subunit | 98.3 | 0.0 | 141.6 | 1 | 0 | 2 | 2.5 | 0.0 | 4.4 | 51.08 | 4 |
| 12 | PSPTO_4867 | phosphoribosylamine--glycine ligase | 89.7 | 0.0 | 120.6 | 1 | 0 | 2 | 3.5 | 0.0 | 6.3 | 45.52 | 5 |
| 13 | PSPTO_4941 | GTP-binding protein HflX | 88.9 | 0.0 | 42.3 | 1 | 0 | 1 | 3.2 | 0.0 | 2.1 | 48.71 | 4 |
| 14 | PSPTO_4800 | glutamate-1-semialdehyde-2,1-aminomutase | 87.1 | 0.0 | 69.7 | 1 | 0 | 1 | 3.3 | 0.0 | 3.3 | 45.38 | 0 |
| 15 | PSPTO_2756 | outer membrane efflux protein | 81.2 | 0.0 | 250.3 | 1 | 0 | 3 | 4.3 | 0.0 | 9.4 | 53.01 | 5 |
| 16 | PSPTO_4898 | heat shock protein YegD | 80.8 | 0.0 | 61.9 | 2 | 0 | 1 | 4.8 | 0.0 | 2.1 | 46.41 | 4 |
| 17 | PSPTO_5601 | ATP synthase subunit alpha | 52.6 | 0.0 | 146.6 | 1 | 0 | 2 | 2.7 | 0.0 | 4.1 | 55.32 | 4 |
| 18 | PSPTO_2379 | translation initiation factor IF-3 | 52.0 | 0.0 | 39.0 | 1 | 0 | 1 | 5.6 | 0.0 | 5.6 | 20.04 | 2 |
| 19 | PSPTO_4560 | outer membrane porin | 50.0 | 0.0 | 106.6 | 1 | 0 | 2 | 3.1 | 0.0 | 4.6 | 50.66 | 2 |
| 20 | PSPTO_1326 | cytochrome o ubiquinol oxidase subunit I | 35.5 | 0.0 | 55.4 | 1 | 0 | 1 | 1.8 | 0.0 | 1.5 | 74.48 | 11 |
| 21 | PSPTO_3885 | tail-specific protease | 35.1 | 0.0 | 63.5 | 1 | 0 | 1 | 2.3 | 0.0 | 2.3 | 77.25 | 5 |

## Supplementary Table 2. Primers used in this study.

| Name | Sequence (5'-3') | Comments |
|---|---|---|
| SAP1-TOPO_F | CACCATGGCGTCTTCTCGAATCATCAT | SAP1 cloning in pENTR/D-Topo vector |
| SAP1-SS_TOPO_F | CACCATGGCCCAACCATCTTTCCGGCCC | SAP1/SAP1ΔSP cloning in pENTR/D-Topo vector |
| SAP1-TOPO_R | AGCAGTGGAAGTGAAATTAAAG | SAP1ΔSP cloning in pENTR/D-Topo vector |
| SAP2-TOPO_F | CACCATGGCGCCTTCTCCGATCATCT | SAP2 cloning in pENTR/D-Topo vector |
| SAP2-SS_TOPO_F | CACCATGGCTCAAACACCTTTCCGTCCC | SAP2/SAP2ΔSP cloning in pENTR/D-Topo vector |
| SAP2-TOPO_R | AGCAGTGGAAGTGAAATTGAAG | SAP2ΔSP cloning in pENTR/D-Topo vector |
| SAP1pro-TOPO_F | CACCTTTCACATTAGTCATCCGGTTT | SAP1 promoter cloning in pENTR/D-Topo vector |
| SAP1pro-TOPO_R | TGTTAGAGATGAATGAGGAATG | SAP1 promoter cloning in pENTR/D-Topo vector |
| SAP2pro-TOPO_F | CACCGCAGTTATCCGGTAACTAGC | SAP2 promoter cloning in pENTR/D-Topo vector |
| SAP2pro-TOPO_R | TGTTAGAGATGACGATGTTGAGTT | SAP2 promoter cloning in pENTR/D-Topo vector |
| SALK_062079_LP | GTGCGTGTTTGTGTGTACGAG | Genotyping of T-DNA insertion plant |
| SALK_062079_RP | TCACGACATGAATGTCATTTTG | Genotyping of T-DNA insertion plant |
| SAIL_646_E08_LP | TTCCAGATAACTCCATCACCG | Genotyping of T-DNA insertion plant |
| SAIL_646_E08_RP | AGAAATGATTCCATTGGCTCC | Genotyping of T-DNA insertion plant |
| Salk_LBb1.3 | ATTTTGCCGATTTCGGAAC | Genotyping of T-DNA insertion plant |
| SAIL_LB1 | GCCTTTTCAGAAATGGATAAATAGCCTTGCTTCC | Genotyping of T-DNA insertion plant |
| SAP2_RNAi_F | CACCGAGAACTCTGGGTCGACTGC | Generation of SAP2 RNAi plant |
| SAP2_RNAi_R | CTTGTGAAAACGCAGAAGCA | Generation of SAP2 RNAi plant |
| GFP_Topo_F | CACCATGgtgagcaagggcgaggag | GFP cloning in pENTR/D-Topo vector |
| GFP_Topo_R | TCAttgtttgcctccctgctg | GFP cloning in pENTR/D-Topo vector |
| MucD_TOPO_F | CACCATGTCGACACCACGCATGAAATC | MucD cloning in pENTR/D-Topo vector |
| MucD_TOPO_R | TTCCGACAGTTTGAAGGTAATGT | MucD cloning in pENTR/D-Topo vector |
| MucD_FlankA_F | CGCGGATCCtgaagaagtaagcacccgaaa | Generation of Pto ΔMucD mutant |
| MucD_FlankA_R | ACATGCATGCacatatggctcccgttcgta | Generation of Pto ΔMucD mutant |
| MucD_FlankB_F | ACATGCATGCtgtcggaataacaggcaggt | Generation of Pto ΔMucD mutant |
| MucD_FlankB_R | CCCAAGCTTcactcacggatcaacactcaa | Generation of Pto ΔMucD mutant |
| pCPP5209_FRT_F | ACATGCATGC GTGTAGGCTGGAGCTGCTTC | Generation of Pto ΔMucD mutant |
| pCPP5209_FRT_R | ACATGCATGC CATATGAATATCCTCCTTA | Generation of Pto ΔMucD mutant |
| MucD_pro_TopoF | CACCTgaagaagtaagcacccgaaa | Generation of Pto ΔMucD mutant |
| Flank_AB_Link_F | AGCCATATGGCATGCTGTCGGAATAACAGGCAGG | Generation of Pto ΔMucD mutant |
| Flank_AB_Link_R | TTCCGACAGCATGCCATATGGCTCCCGTTCGT | Generation of Pto ΔMucD mutant |
| FlankA_GeneArt_F | GTGATATCGAGCTCGTGCGGATCCTGAAGAAGTAAGCACCC | Generation of Pto ΔMucD mutant |
| FlankA-Gm_F | GCATGCATGTTACGCAGCAGCAACGAT | Generation of Pto ΔMucD mutant |
| FlankA-Gm_R | GCGTAACATGCATGCATGGCTCCCGTTCGT | Generation of Pto ΔMucD mutant |
| Gm-FlankB_F | TAAGCATGCCAGGCAGGTTTGATCGGGAAGG | Generation of Pto ΔMucD mutant |
| Gm-FlankB_R | CTGGCATGCTTAGGTGGCGGTACTTGGGTCGA | Generation of Pto ΔMucD mutant |
| SAP1_D63A_F | CCCCGCTTCCGTCGTATTCGCACTCGGTGGTCGAGA | SAP1 site mutagenesis constrution |
| SAP1_D63A_R | GAATTCTCGACCACCGAGTGCGAATACGACGGAACG | SAP1 site mutagenesis constrution |
| SAP1_D136A_F | GAATTTGCTTTAGCAGTTGTGTCTATCCAGTCC | SAP1 site mutagenesis constrution |
| SAP1_D136A_R | GATAGACACAACTGCTAAAGCAAATTCGCCCGAG | SAP1 site mutagenesis constrution |

| | | |
|---|---|---|
| SAP1_D39A_F | CAACCGTCATCAACCAACGCACACCTCTCGTCC | SAP1 site mutagenesis constrution |
| SAP1_D39A_R | GGAAGCGGGGACGAGAGGTGTGCGTTGGTTGATG | SAP1 site mutagenesis constrution |
| SAP1_D72A_F | GTATTCGACCTCGGTGGTCGAGCATTCTGGGTCG | SAP1 site mutagenesis constrution |
| SAP1_D72A_R | TTGGTCACAGTCGACCCAGAATGCTCGACCACCG | SAP1 site mutagenesis constrution |
| SAP1_D136A_F | ACCTCGGGCGAATTTGCTTTAGCAGTTGTGTCTATC | SAP1 site mutagenesis constrution |
| SAP1_D136A_R | GAATTTGCTTTAGCAGTTGTGTCTATCCAGTCCACT | SAP1 site mutagenesis constrution |
| SAP1_D290A_F | AATAGGAGGAACCAAAATCAGCGCCGTCAATCCTT | SAP1 site mutagenesis constrution |
| SAP1_D290A_R | CAACACCGTGTAAGGATTGACGGCGCTGATTTTGG | SAP1 site mutagenesis constrution |
| SAP1_D382A_F | GTCATCTGTTTGGGTTTCGTTGCAGGAGGAGTC | SAP1 site mutagenesis constrution |
| SAP1_D382A_R | GGCTCCTGGGTTGACTCCTCCTGCAACGAAACCC | SAP1 site mutagenesis constrution |
| SAP1_D400A_F | ATCGGAGGGTTCCAGTTGGAGGCTAATTTGATCG | SAP1 site mutagenesis constrution |
| SAP1_D400A_R | CAAATCAAATTCGATCAAATTAGCCTCCAACTGG | SAP1 site mutagenesis constrution |
| SAP1_D406A_F | GGAGGATAATTTGATCGAATTTGCTTTGGCGAGT | SAP1 site mutagenesis constrution |
| SAP1_D406A_R | CCCAAATTTGTTACTCGCCAAAGCAAATTCGATC | SAP1 site mutagenesis constrution |
| CrubASP1_qPCR_F | CAAATTCGCCGTGTGTCTTA | qPCR_analysis |
| CrubASP1_qPCR_R | TCCGAGGATTTTTCACCTTG | qPCR_analysis |
| CrubASP2_qPCR_F | ACTTGCGGCTCTATTCCAGA | qPCR_analysis |
| CrubASP2_qPCR_R | CATTGCAGCCATACCAACTG | qPCR_analysis |
| EsalASP_qPCR_F | CGGCATATGCTTCTCTCCTC | qPCR_analysis |
| EsalASP_qPCR_R | CCCGCAACTGAAAACAAAAT | qPCR_analysis |
| AlyrASP1_qPCR_F | TGCGGATCAACCTCTCTTCT | qPCR_analysis |
| AlyrASP1_qPCR_R | GGGGAGGAAAACGTAAGGTC | qPCR_analysis |
| AlyrASP2_qPCR_F | GAGAACTCTGGGTCGACTGC | qPCR_analysis |
| AlyrASP2_qPCR_R | CGCCGCAAGTGTTGTTACTA | qPCR_analysis |
| SolycASP_qPCR_F | TGATCTTGGTGGCCAATTTT | qPCR_analysis |
| SolycASP_qPCR_R | TCCCAGTAACCGTGTTGTCA | qPCR_analysis |
| BraACT2_qPCR_F | GACCTTTAACTCTCCCGCTATGT | qPCR_analysis |
| BraACT2_qPCR_R | CCATCACCAGAATCCAGCACA | qPCR_analysis |
| SAP2_Crispr1_F | ATTGGTAGCGTCGGTGAAACCGTT | SAP2 crisper cas9 construction |
| SAP2_Crispr1_R | AAACAACGGTTTCACCGACGCTAC | SAP2 crisper cas9 construction |
| SAP2_Crispr2_F | ATTGGGAGTTCGTTAAACAAGCAG | SAP2 crisper cas9 construction |
| SAP2_Crispr2_R | AAACCTGCTTGTTTAACGAACTCC | SAP2 crisper cas9 construction |
| MucD_F106Y_F | GCTCAGTCGTTGGGTTCGGGCTATATCATCTCCCCC | MucD site mutagenesis constrution |
| MucD_F106Y_R | GCCATCGGGGGAGATGATATAGCCCGAACCCAA | MucD site mutagenesis constrution |
| MucD_S394A_F | CAGCAACCGTCTGGGCGTTGCGGTGATCGAGCTG | MucD site mutagenesis constrution |
| MucD_S394A_R | CGGCGGTCAGCTCGATCACCGCAACGCCCAGACG | MucD site mutagenesis constrution |

**Supplementary References**

1. Letunic I, Bork P. Interactive tree of life (iTOL) v3: an online tool for the display and annotation of phylogenetic and other trees. *Nucleic Acids Res* **44**, W242-W245 (2016).

2. Zimmermann P, Hirsch-Hoffmann M, Hennig L, Gruissem W. GENEVESTIGATOR. Arabidopsis microarray database and analysis toolbox. *Plant Physiol* **136**, 2621-2632 (2004).
