# Supplementary material for: Site-specific cleavage of bacterial MucD by secreted proteases mediates antibacterial resistance in Arabidopsis
Source: Nat Commun. 2019 Jun 28;10:2853. doi: 10.1038/s41467-019-10793-x (PMC6599210; doi:10.1038/s41467-019-10793-x)
Supplement: Supplementary file 3 — Description of Additional Supplementary Files [file 41467_2019_10793_MOESM3_ESM.pdf]

Description of Additional Supplementary Files

File Name: Supplementary Data 1
Description: Protein sequences showing best BLAST hits to Pto DC3000 MucD in selected organisms including bacteria, plants, animals, oomycetes, and fungi.
