# Supplementary material for: Site-specific cleavage of bacterial MucD by secreted proteases mediates antibacterial resistance in Arabidopsis
Source: Nat Commun. 2019 Jun 28;10:2853. doi: 10.1038/s41467-019-10793-x (PMC6599210; doi:10.1038/s41467-019-10793-x)
Supplement: Supplementary file 5 — Reporting Summary [file 41467_2019_10793_MOESM5_ESM.pdf]

nature research

Corresponding author(s): Kenichi Tsuda

Last updated by author(s): May 31, 2019

# Reporting Summary

Nature Research wishes to improve the reproducibility of the work that we publish. This form provides structure for consistency and transparency in reporting. For further information on Nature Research policies, see Authors & Referees and the Editorial Policy Checklist .

## Statistics

For all statistical analyses, confirm that the following items are present in the figure legend, table legend, main text, or Methods section.

| n/a | Confirmed | |
|---|---|---|
| [ ] | [x] | The exact sample size (*n*) for each experimental group/condition, given as a discrete number and unit of measurement |
| [ ] | [x] | A statement on whether measurements were taken from distinct samples or whether the same sample was measured repeatedly |
| [ ] | [x] | The statistical test(s) used AND whether they are one- or two-sided<br>*Only common tests should be described solely by name; describe more complex techniques in the Methods section.* |
| [x] | [ ] | A description of all covariates tested |
| [ ] | [x] | A description of any assumptions or corrections, such as tests of normality and adjustment for multiple comparisons |
| [ ] | [x] | A full description of the statistical parameters including central tendency (e.g. means) or other basic estimates (e.g. regression coefficient) AND variation (e.g. standard deviation) or associated estimates of uncertainty (e.g. confidence intervals) |
| [ ] | [x] | For null hypothesis testing, the test statistic (e.g. *F*, *t*, *r*) with confidence intervals, effect sizes, degrees of freedom and *P* value noted<br>*Give P values as exact values whenever suitable.* |
| [x] | [ ] | For Bayesian analysis, information on the choice of priors and Markov chain Monte Carlo settings |
| [x] | [ ] | For hierarchical and complex designs, identification of the appropriate level for tests and full reporting of outcomes |
| [x] | [ ] | Estimates of effect sizes (e.g. Cohen's *d*, Pearson's *r*), indicating how they were calculated |

*Our web collection on statistics for biologists contains articles on many of the points above.*

## Software and code

Policy information about availability of computer code

| | |
|---|---|
| Data collection | ZEN 2009 Light Edition (http://www.zeiss.com.cn/microscopy/downloads/zen.html)<br>MEROPS (http://merops.sanger.ac.uk)<br>TAIR10 (https://www.arabidopsis.org/tools/index.jsp) |
| Data analysis | Excel 2016 (https://products.office.com/en-us/excel)<br>R 3.4.3 (https://cran.r-project.org/bin/windows/base/old/3.4.3/)<br>ImageJ (https://imagej.nih.gov/ij/)<br>iTOL (https://itol.embl.de/)<br>CLUSTALW (http://www.genome.jp/tools/clustalw/)<br>SignalP4.1 (http://www.cbs.dtu.dk/services/SignalP-4.1/) |

For manuscripts utilizing custom algorithms or software that are central to the research but not yet described in published literature, software must be made available to editors/reviewers. We strongly encourage code deposition in a community repository (e.g. GitHub). See the Nature Research guidelines for submitting code & software for further information.

## Data

Policy information about availability of data

All manuscripts must include a data availability statement. This statement should provide the following information, where applicable:
- Accession codes, unique identifiers, or web links for publicly available datasets
- A list of figures that have associated raw data
- A description of any restrictions on data availability

All data underlying the findings are available from the corresponding author upon request.

# Field-specific reporting

Please select the one below that is the best fit for your research. If you are not sure, read the appropriate sections before making your selection.

☒ Life sciences ☐ Behavioural & social sciences ☐ Ecological, evolutionary & environmental sciences

For a reference copy of the document with all sections, see nature.com/documents/nr-reporting-summary-flat.pdf

# Life sciences study design

All studies must disclose on these points even when the disclosure is negative.

| | |
|---|---|
| Sample size | Sample sizes were determined according to common practice in the field and prior experience that allow statistical significance to be tested. |
| Data exclusions | No data were excluded from the analyses. |
| Replication | All experiments was subjected to at least three independent biological experiments with similar results. |
| Randomization | Different plant genotypes and bacterial strains were used in this study. Different plant genotypes grown in soils were randomly placed in growth chamber to ensure equal growth conditions. For bacterial experiments, different strains in culture tubes with different treatment were randomly placed in culture chamber. |
| Blinding | Blinding was not applied. |

# Reporting for specific materials, systems and methods

We require information from authors about some types of materials, experimental systems and methods used in many studies. Here, indicate whether each material, system or method listed is relevant to your study. If you are not sure if a list item applies to your research, read the appropriate section before selecting a response.

## Materials & experimental systems

| n/a | Involved in the study | |
|---|---|---|
| ☐ | ☒ | Antibodies |
| ☒ | ☐ | Eukaryotic cell lines |
| ☒ | ☐ | Palaeontology |
| ☒ | ☐ | Animals and other organisms |
| ☒ | ☐ | Human research participants |
| ☒ | ☐ | Clinical data |

## Methods

| n/a | Involved in the study | |
|---|---|---|
| ☒ | ☐ | ChIP-seq |
| ☒ | ☐ | Flow cytometry |
| ☒ | ☐ | MRI-based neuroimaging |

## Antibodies

**Antibodies used**

Anti-GFP in Rabbit (Abcam, Cat#AB6556, Lot#GR136046_1);
Anti-HA in Rat (Roche, Cat#11867423001, Lot#11608200);
Anti-flag in mouse (Sigma-Aldrich, Cat#F3165, Lot#SLBT6752);
Anti-GST in mouse (Virogen, Cat#101-A-100, Lot#GS-D8/G2-60);
Anti-His in goat (ThermoFisher, Cat#PA1-23024, Lot#RG2230117);
Anti-rabbit HRP in goat (Santa Cruz, Cat#sc-2004),
Anti-rat HRP in goat (Santa Cruz, Cat#sc-2006);
Anti-mouse HRP in sheep (GE Healthcare Life Science; Cat#NA931)
Anti-goat HRP in donkey (Santa Cruz, Cat#sc-2020)
Anti-PR1 (kindly provided by Prof. Xinnian Dong);

**Validation**

Anti-GFP in Rabbit validation information, the polyclonal antibody was generated with full length protein corresponding to GFP, which could detect variants of Aequorea victoria GFP, such as RS-GFP, YFP, eGFP. It has been validated in associated papers. Application Ref., https://www.abcam.com/gfp-antibody-ab6556.html

Anti-HA in Rat validation information, the monoclonal antibody was generated with HA peptide (YPYDVPDYA) derived from the influenza hemagglutinin protein. It has been validated in associated papers. Application Ref., https://www.sigmaaldrich.com/catalog/product/roche/roahaha?lang=de&region=DE

Anti-flag in mouse validation information, the monoclonal antibody was generated with FLAG peptide (DYKDDDDK) derived from the influenza hemagglutinin protein. It has been validated in associated papers. Application Ref., https://www.sigmaaldrich.com/catalog/product/roche/roahaha?lang=de&region=DE

Anti-GST in mouse validation information, the monoclonal antibody was generated with purified glutathione. It has been validated in associated papers. Application Ref., https://www.virogen.com/products/anti-glutathion-monoclonal-antibody-igg1

Anti-His in goat validation information, the polyclonal antibody was generated with 6xHis peptide (HHHHHH). It has been validated in associated papers. Application Ref., https://www.thermofisher.com/antibody/product/6x-His-Tag-Antibody-Polyclonal/PA1-23024

Anti-mouse HRP in sheep validation information, the secondary antibody conjugated to HRP has been validated in associated papers. Application Ref., https://www.gelifesciences.com/en/us/shop/protein-analysis/blotting-and-detection/blotting-standards-and-reagents/amersham-ecl-hrp-conjugated-antibodies-p-06260#tech-spec-table

Anti-rabbit HRP in goat, anti-rat HRP in goat, and anti-goat IgG in donkey validation information, the secondary antibody conjugated to HRP has been validated in associated papers. Data unavailable due to collapse of Santa Cruz.

Anti-PR1 in rabbit validation information, the polyclonal antibody was generated with synthetic peptide against C-terminus of Arabidopsis PR1 protein. Application Ref. Kinkema et al., Plant Cell, 2000, 12(12):2339-2350.
